# Sequence Expression of Supernumerary B Chromosomes: Function or Fluff?

**DOI:** 10.3390/genes10020123

**Published:** 2019-02-08

**Authors:** Elena Dalla Benetta, Omar S. Akbari, Patrick M. Ferree

**Affiliations:** 1 W. M. Keck Science Department of Claremont McKenna, Pitzer, and Scripps Colleges, Claremont, CA 91711, USA; edallabenetta@ucsd.edu; 2 Division of Biological Sciences, Section of Cell and Developmental Biology, University of California, San Diego, La Jolla, CA 92093, USA; oakbari@ucsd.edu

**Keywords:** B chromosomes, PSR (Paternal sex ratio), genome elimination, ncRNAs (non coding RNAs), selfish elements, super-Mendelian, repeated elements

## Abstract

B chromosomes are enigmatic heritable elements found in the genomes of numerous plant and animal species. Contrary to their broad distribution, most B chromosomes are non-essential. For this reason, they are regarded as genome parasites. In order to be stably transmitted through generations, many B chromosomes exhibit the ability to “drive”, i.e., they transmit themselves at super-Mendelian frequencies to progeny through directed interactions with the cell division apparatus. To date, very little is understood mechanistically about how B chromosomes drive, although a likely scenario is that expression of B chromosome sequences plays a role. Here, we highlight a handful of previously identified B chromosome sequences, many of which are repetitive and non-coding in nature, that have been shown to be expressed at the transcriptional level. We speculate on how each type of expressed sequence could participate in B chromosome drive based on known functions of RNA in general chromatin- and chromosome-related processes. We also raise some challenges to functionally testing these possible roles, a goal that will be required to more fully understand whether and how B chromosomes interact with components of the cell for drive and transmission.

## 1. Introduction

Since the time when microscopy first allowed visualization of hereditary material, researchers have observed peculiar chromosome variants in the genomes of higher eukaryotes. For any given species, the core of the genome consists of a certain number of A chromosomes, which carry all genes needed collectively for the organism’s development, metabolism, and reproduction. Thus, without the complete set of A chromosomes, the organism cannot survive. However, extra or supernumerary non-essential chromosomes, termed B chromosomes, have been detected in numerous plant and animal species [1,2,3,4]. The frequency of B chromosomes within a given population can range from very low to complete fixation among all individuals, and a carrying individual can contain one to as many as ten or more B chromosome copies in each nucleus [1,2,3]. Interestingly, with little exception, B chromosomes are not essential for the organism. Indeed, the fact that B chromosomes can persist without providing any measurable benefit has contributed to the view that they are genome parasites [5]. But if they do not help the organism, how then do B chromosomes persist, thereby defying the expectation that non-essential genetic elements are eventually lost?

Previous work in various B-carrying organisms has provided some insights regarding this question. For example, in several grasshopper species, certain B chromosomes are transmitted to progeny through both parents, and they segregate with very high efficiency to daughter cells during mitotic and meiotic divisions [6,7]. In these organisms, the B chromosomes appear to behave similarly to the A chromosomes. In contrast, B chromosomes in other organisms exhibit “drive”—that is, they behave in specific ways that defy normal Mendelian transmission patterns in order to ensure their inheritance in subsequent generations [8]. For example, one B chromosome in a rye species counters its tendency to loss through a form of mitotic drive. Specifically, the sister B chromatids fail to separate (i.e., non-disjoin) in the division that produces the vegetative (non-gametic) cell and pollen (gametic) cell [9,10]. Moreover, the unseparated B chromatids tend to segregate toward the side of the spindle that will give rise to the pollen cell. This tropism of the B chromatid pair for the future pollen cell, which can lead to an accumulation of multiple B chromosome copies in offspring, is thought to occur by the B chromatids utilizing an inherent asymmetry in the makeup of the spindle apparatus [9]. Indeed, B chromosomes may tend to drive in this way in plants because of the universally asymmetric spindle at the pollen production stage [11]. 

As a remarkably different example of drive, the jewel wasp *Nasonia vitripennis* harbors a B chromosome known as PSR (for Paternal Sex Ratio) that is transmitted via the sperm (i.e., paternally) to progeny [12]. Interestingly, this B chromosome causes complete loss of the sperm’s hereditary material but not itself during the first mitotic division of the embryo [13]. Interestingly, the PSR chromosome does not eliminate itself, but instead associates with the functional egg-derived chromosomes, and successfully segregates with them. Due to the fact that in wasps and other hymenopteran insects, males normally develop as haploids from unfertilized eggs while females develop as diploids from fertilized eggs, this genome elimination event converts fertilized embryos, which should become female, into haploid B chromosome-carrying males, thereby ensuring B chromosome transmission.

Even though we know what happens at the descriptive level in these cases of drive, what remains to be determined is how mechanistically B chromosomes drive in their resident genomes. Two general possibilities exist: a B chromosome may either (i) act passively, being transmitted and/or driving due to the intrinsic properties of its own DNA sequences [14], or (ii) it may operate actively through expression of its DNA sequences [9,15]. Here, we focus on the second possibility by reviewing a number of different types of B chromosome-linked DNA sequences, many of which are repetitive and non-coding in nature, that have been identified through genetic and genomic analyses. We highlight a subset of these DNA sequences that are known to be expressed, and we propose possible roles for each. Several recent studies have demonstrated that B chromosomes can influence the expression of A-linked genes [16,17,18,19] and epigenetic marks of A chromatin, reviewed in [20,21]. However, given the non-essential nature of most B chromosomes, these effects may not substantially affect the biology of the organism. Thus, we limit our attention here to B-expressed sequences and their potential roles in B chromosome drive and transmission. Finally, we raise some challenges to functionally testing these possible roles, a goal that will be required to more fully understand whether and how B chromosomes interact with components of the cell.

## 2. B Chromosomes Are Mosaics of Protein-Coding and Repetitive, Non-Coding Sequences

Genetic and genomic studies have been performed in a number of different organisms, including but not exclusive to maize [22], rye [19,23], grasshoppers [24], wasps [25,26], cichlids [27,28], raccoon dogs [29] and fungi [30,31], in order to identify specific DNA sequences carried by B chromosomes (see Table 1). The repertoire of B-linked DNA sequences includes both protein-coding and non-coding sequences. The origin of any given B-linked sequence may date back to the beginning of the B chromosome itself, or the sequence may have arisen subsequently as a copy of another B-linked gene or of an ancestral gene located on an A chromosome that was moved via transposable element (TE) activity, inter-chromosomal meiotic recombination, or imperfect DNA repair [19].

The majority of known B-linked protein-coding genes match genes located on the A chromosomes and belong to nearly all protein function categories [19,29,32,33,34,35]. Most B-linked protein-coding genes are degenerate; they can be present as partial gene copies, such as truncated forms or missing exons, or they can show low sequence similarity across their entire lengths to the ancestral sequences [35]. For this reason, many B-linked protein-coding genes are considered to be pseudogenes [19]. The few B-linked protein-coding genes that do show high sequence similarity to their ancestral copies are likely not needed for the organism because the B chromosomes themselves are non-essential. Thus, given the lack of functional constraint on such B-linked protein-coding genes, high sequence similarity may indicate that the origin of the B-linked copy from its ancestral gene was a relatively recent event.

Despite the presence of protein-coding sequences, it appears that most B chromosomes consist primarily of non-coding sequences including TEs, simple satellites, and complex satellite-like repeats [3,36,37,38,39,40]. Such highly repetitive DNA sequences are known to be enriched in the heterochromatin that surrounds the centromeres of the A chromosomes [40]. For this reason, it has been proposed that B chromosome formation begins with duplication of an A chromosome followed by the loss of its euchromatic chromosome arms, thus producing a nascent B chromosome consisting mainly of a centromere and its pericentromeric regions. Over time, TE activity can move genes from the A chromosomes onto the B chromosome; these sequences may then undergo mutational decay, further duplication through replication slippage, or rearrangement events such as intra-chromosomal inversion, deletion, and translocation [40]. We should mention here that the PSR chromosome present in the jewel wasp contains transposon-like sequences that appear to be absent from the wasp genome but to match sequences present in another wasp species [41,42]. Such patterns open up the possibility that this B chromosome derives from a chromosome of another species that moved into the wasp genome through interspecific hybridization or by parasites or food sources [43,44,45,46].

## 3. Expression of B-Linked DNA Sequences

Despite our current knowledge of some DNA sequences carried by B chromosomes, only a handful of studies have addressed which of them are expressed. Certainly, transcription of any given DNA sequence alone does not guarantee that it is functional; work in different model organisms suggests that much of the non-coding part of the A genome may be transcribed without function [47]. Nevertheless, a reasonable (and perhaps obvious) assumption is that a locus that is functional will at least be expressed at the RNA level. To date, several studies have identified RNAs produced by B chromosomes, either by examination of individual B-linked sequences or through whole genome approaches such as RNA-Seq. To our knowledge, no individual B-linked sequence has yet been tested for functionality through genetic manipulation. However, all B-linked sequences producing RNA should be considered as potential candidates for involvement in B chromosome dynamics and drive. Here, we highlight several examples of B-linked sequences that are known to express RNAs, and we speculate on the possible functions of these sequences in light of the types of RNAs that they produce and the underlying B chromosome biology in each case.

### 3.1. Copies of Protein-Coding Genes

Of the previously identified B-linked DNA sequences that are copies of A-linked protein-coding genes, few are known to be expressed (Table 1) [19,23,24,27,28,48,49]. For example, a B chromosome in the cichlid fish *Astatotilapia latifasciata* was shown to express multiple different protein-coding genes. Three of these expressed genes derive from the ancestral A-linked genes encoding Separin, Tubulin B1 (*TUBB1*), and *KIF11* [27,28]. Interestingly, these A-linked genes play important roles in chromosomal segregation during cell division: *TUBB1* is involved in microtubule organization [50], *KIF11* functions in centrosome behavior and spindle assembly [50], and Separin mediates the release of sister chromatids at the onset of anaphase [50]. It has been proposed that because these genes are implicated in different aspects of chromosome segregation, expression of the B-linked variants may somehow promote B chromosome transmission such that they are inherited to over 50% of the gametes [27,28]. Indeed, a number of B-linked genes that derive from protein-coding genes involved in various aspects of the cell cycle and cell division have been detected in other organisms [19,24,29,34]. However, in most of these other cases, it is not yet known which of the protein-coding gene variants are expressed.

We point out here an important consideration: that any expressed protein-coding gene that affects aspects of cell division would likely impact the A chromosomes in addition to the B chromosome, potentially having large costs to the organism. Thus, proteins that play roles in B chromosome drive may be expected to specifically affect the B chromosome. Certain chromatin-associated proteins, which could have affinity for specific DNA sequences found uniquely on B chromosomes, would fulfill such an expectation. This idea is consistent with previously proposed models invoking co-evolving centromere repeats and their chromatin proteins as agents of centromere/meiotic drive [51]. Copies of histones H3 and H4 are known to be expressed from B chromosomes in different grasshopper species [52,53]. However, it remains to be determined whether variants of these conventional histones or other non-histone chromatin proteins are carried and expressed by B chromosomes. 

### 3.2. Transposable Elements

DNA TEs, retro-TEs, and TE-like elements are abundant in higher eukaryotes, making up anywhere from ~5 to as much as 50% of a given eukaryotic genome [54,55]. It is, therefore, no surprise that these elements have been found to be carried and expressed by B chromosomes in a number of organisms including rye [15,33], maize [56], fishes [39,57], and the jewel wasp [41,42]. It is difficult to imagine a scenario in which TE expression per se could enhance B chromosome drive. Moreover, a substantial amount of cellular energy is devoted to the silencing of TE expression, and unsilenced TEs can lead to severe genome instability [58]. However, TEs may play secondary but important roles in B chromosome drive. Given the potential of TEs to mobilize and amplify within single generations, it is expected that these genetic elements move frequently between the A and B chromosomes over short periods of evolutionary time; as mentioned above, such movement likely serves as a mechanism for the transfer of gene copies between the A and B chromosomes [39,40]. Additionally, TEs that have moved onto B chromosomes may themselves degenerate and become pseudogenes, they may fuse with non-TE sequence to form new genes [39,40], or they may decay over time and become tandemly copied to form arrays of complex satellite-like repeats [59]. Any such TE-derived sequence may itself be expressed through the transcriptional regulatory sequences of transposase or other TE-associated genes, or it may induce the expression of adjacent sequences that would otherwise be transcriptionally silent.

### 3.3. Long Non-Coding RNAs

An interesting class of candidates for involvement in B chromosome transmission and drive consists of long non-coding RNAs (lncRNAs). Bioinformatically, lncRNAs are challenging to identify from RNA expression datasets for a number of reasons. For one, it is difficult to identify secondary structural domains that suggest potential function of a putative lncRNA. Additionally, long RNAs that function as structural molecules may contain cryptic, unused open reading frames, leading to ambiguity in bioinformatically assigning such RNAs as coding or non-coding. Despite these challenges, previous work has led to the identification of putative lncRNAs expressed from B chromosomes in the jewel wasp and in cichlids [25,60]. 

In the jewel wasp, comparison of testis transcriptomes between wild type and B chromosome-carrying (PSR+) males led to the identification of ten transcripts, ranging between ~500–1500 nucleotides in length, that are present only in the PSR + genotype [25,26]. These transcripts represent the highest-expressed sequences from the PSR chromosome. Fluorescence in situ hybridization (FISH) and PCR of genomic DNA were used to demonstrate that the cognate DNA sequences of these transcripts are located exclusively on the PSR chromosome [25,26]. A couple of these transcripts contain potential, short open reading frames, but the majority of them were bioinformatically predicted to be non-coding [25]. A different study in the cichlid *A. latifasciata* identified a transcript corresponding to a non-coding DNA repeat represented in multiple copies on a B chromosome in this organism [60]. This transcript was shown to be expressed in multiple different fish tissues [60].

A central question is whether such non-coding RNAs are functional, especially with regard to B chromosome drive. While no studies have yet demonstrated functionality of these RNAs, some interesting speculations stem from examples of lncRNA function in non-B systems. It has become apparent that lncRNAs span a wide range of cellular and developmental processes [61,62], but those of particular interest here pertain to chromatin and chromosome dynamics. In particular, two well-studied groups of lncRNA pertain to the X chromosome. In the fruit fly *Drosophila melanogaster* the roX1 and roX2 lncRNAs associate with the male-specific lethal (MSL) proteins to form the dosage compensation complex (DCC) in young male embryos [63]. This complex localizes to “entry” sites located along the male’s single X chromosome. There, the DCC spreads to other regions on the X, where it ultimately induces transcriptional upregulation of most X-linked genes [64]. This effect involves remodeling of X chromatin through acetylation of Lysine residue 16 of histone H4 (H4K16ac) and phosphorylation of Serine residue 10 of histone H3 (H3S10p), each by a different enzymatic activity of DCC-associated components (reviewed in [65]). In this case, the roX lncRNAs play an indispensable role as a structural “glue” that scaffolds together the DCC proteins [66]. In mammals, a different lncRNA known as Xist is expressed initially from both X chromosomes during early embryogenesis but its expression is eventually turned off on one of the two X chromosomes (reviewed in [67]). Xist coats the X chromosome that continues to express it, an effect that leads to the association of the Polycomb Repressive Complex (PRC) and its trimethylation of Lysine residue 27 of histone H3 (H3K27me3). This histone mark leads to the facultative heterochromatinization of this Xist-expressing X chromosome, leaving the other X in a transcriptionally active state [68]. lcnRNA function is not limited to the X chromosome; these molecules also facilitate chromatin remodeling elsewhere in the genome, and they function in other aspects of chromatin dynamics (reviewed in [69,70]). 

**Figure 1:**
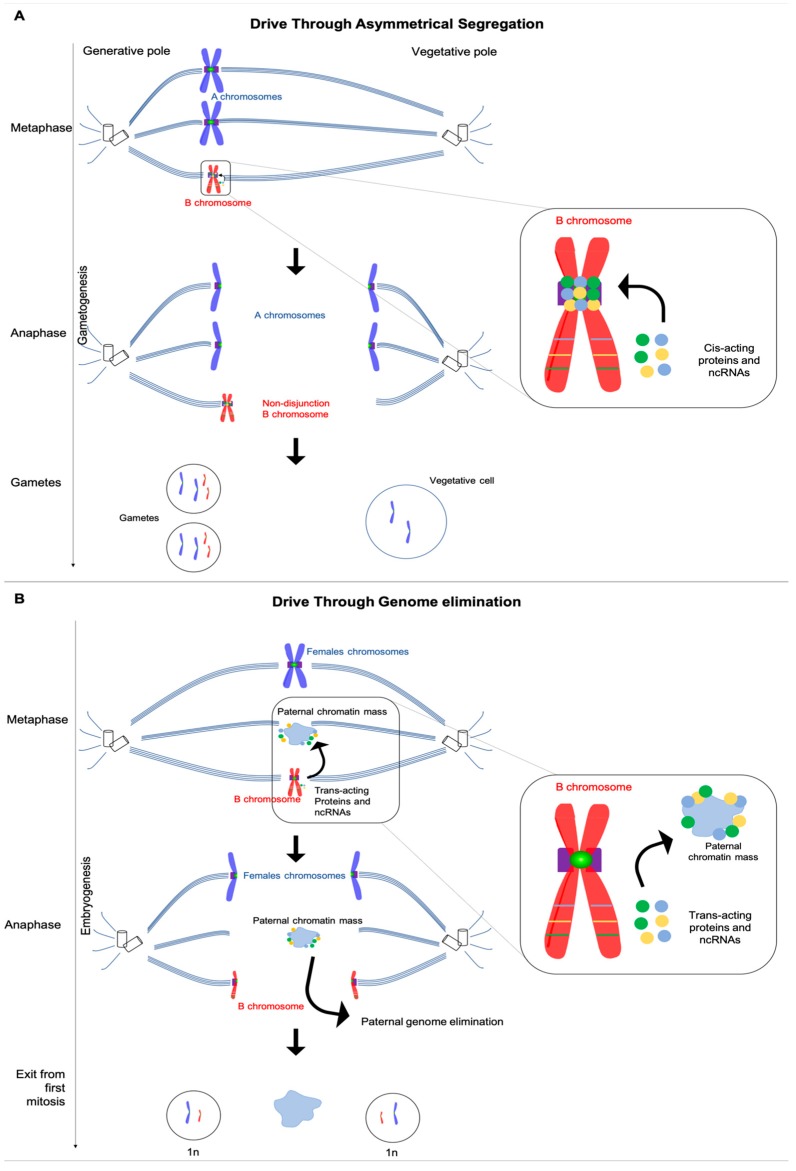
Possible roles for B-expressed sequences in B drive. (**a**) B chromosome drive through asymmetrical segregation and non-disjunction could involve cis-acting B specific products (proteins or ncRNAs) that retard release of the two sister chromatids at the kinetochore. The sister B chromatid pair then migrates preferentially toward the generative pole due to an intrinsic asymmetry of the spindle apparatus. As a result, multiple copies of the B chromosome accumulate in progeny over multiple generations. (**b**) B chromosome drive through genome elimination, such as occurs by the PSR chromosome in the jewel wasp *Nasonia vitripennis*, occurs during the first mitotic division of the newly fertilized embryo. In this model, B-chromosome-expressed protein or RNA could localize preferentially with the paternal chromatin, recruiting chromatin-remodeling enzymes that disrupt normal chromatin remodeling dynamics through abnormal histone modification. As a consequence, the paternal chromatin forms a condensed mass that is unable to resolve into chromosomes and segregate properly.

Taken together, these examples demonstrate the potential for lncRNAs to associate with specific chromosome regions and not others, as well as their ability to facilitate specific alterations of chromatin. Thus, it is intriguing to speculate that B chromosome-expressed lncRNAs may play unique roles in B chromosome drive in certain cases through such chromatin interactions. For example, in both rye and maize, B chromosomes that are deleted for a small region of repetitive DNA lose their ability to drive by nondisjunction at the pollen mitosis stage [9,10]. Currently it is not known if these repeats are transcribed from the undeleted B chromosomes. However, if they are expressed, then their encoded RNAs could associate with the centromeric regions where they may recruit enzymes that interact with the cohesin machinery. Such an effect could, in turn, retard the separation of the sister B chromatids during anaphase so that both B chromatids end up in the gamete (Figure 1). 

It has been proposed that the putative lncRNAs expressed by the PSR chromosome in the jewel wasp may underlie the elimination of the paternally-inherited half of the wasp genome [26]. Previous work demonstrated that certain histone marks (H3K9me3, H3K27me1, and H4K20me1) appeared in abnormal patterns on the paternal chromatin immediately before its elimination [71]. The abnormal placement of these histone marks may block subsequent chromatin remodeling events, such as histone phosphorylation, that are essential for normal condensation of chromatin into chromosomes during mitosis [71]. An intriguing possibility is that PSR induces the abnormal histone marks through one or more of the identified lncRNAs. For example, one or more of these molecules may associate with the paternal chromatin and recruit chromatin-remodeling enzymes that disrupt normal chromatin dynamics [71]. Regardless of the mechanism, PSR must possess some way of sparing itself from this abnormal chromatin remodeling [71] (Figure 1).

### 3.4. Small Non-Coding RNAs

So far, little is known about the potential for B chromosomes to express small RNAs, non-coding molecules that typically range between ~21–33 nucleotides in length (reviewed in [61]). A multitude of studies have characterized the functional roles of the three major classes of small RNAs and their corresponding pathways: micro-RNAs (miRNAs), which block translation of their cognate mRNA targets, endogenous small interfering RNAs (endo-siRNAs), which inhibit translation by inducing degradation of target mRNAs, and PIWI-associated RNAs (piRNAs), which facilitate the transcriptional silencing of chromatin through the association of certain chromatin-remodeling enzymes (reviewed in [72]). The functions of these different small RNA classes are not completely distinct from one another since there is evidence of some crossover between small RNA pathways [73]. To our knowledge, only one study, conducted in the jewel wasp, has detected small RNAs expressed from a B chromosome [26]. In this insect, several different small RNAs were found to be produced by PSR at expression levels matching those of more abundant small RNAs expressed from the A chromosomes [26]. Interestingly, the most abundant PSR-specific small RNA exhibits peculiar properties, having a length (32–33 nt) and starting in a uracil similar to piRNAs while appearing to be processed from a hairpin precursor like endo-siRNAs [26]. More work will be required to better understand to which class this and other PSR-expressed small RNAs belong. However, this work demonstrates that B chromosomes can, indeed, express this type of non-coding RNA. Additionally, given the link of certain small RNAs in chromatin remodeling, it should be strongly considered that B chromosomes like PSR, whose drive involves chromatin remodeling, may drive at least in part through the actions of small RNAs. 

## 4. Functional Testing of Expressed B Loci and Some Challenges

Previous studies aimed at identifying functional B-specific sequences have been restricted to deletion analysis in rye and maize [9,10], jewel wasp, [14] and grasshoppers [74]. Although certain deletions of B chromosomes elicited a loss of drive, it is still unclear in each of these cases which individual sequence(s) within the deleted regions underlie drive and transmission [9,10,14,74]. Until only recently have studies begun to uncover individual RNAs that are expressed by B-linked sequences. Given that most known B chromosomes are not essential for the organism, it may be that much of B chromosome expression may be nothing more than noise. A fundamental question is whether any B expressed loci are functional, and if so, which ones. The ideas presented here may serve as some basis for deciding which candidate loci to prioritize within each B chromosome system. But one thing is certain: fully understanding if and how a given locus is involved in B chromosome transmission or drive will ultimately require some form of genetic manipulation. Such a goal has been challenging due to the fact that most studied B chromosomes reside in non-model organisms that lack traditional genetic tools. However, the development of CRISPR/Cas9 genome editing has made genetic manipulation of individual loci possible in almost any organism, model or not. In principle, this method promises to allow “knock out” of target loci on B chromosomes or, alternatively, the transgenic expression of B chromosome-derived sequences in a non-B genotype, in order to test for functionality.

Just as there is strong promise for CRISPR/Cas9 in achieving these goals, there are some substantial obstacles that will need to be tackled. For example, unlike essential genes located on the A chromosomes, which provide lethal or semi-lethal phenotypes when altered, mutant alleles created by the editing of B-linked loci would not provide any overt phenotype to follow. Contrarily, any such induced mutant allele that affects B chromosome drive would likely lead to quick loss of the B chromosome under study. Another difficulty would be mutagenesis of candidate sequences that are present in multiple copy number, such as the complex repeats that express putative lncRNAs in the jewel wasp [26]. A less problematic goal may be the expression of candidate B linked sequences from transgenes inserted through CRISPR/Cas9 and homology-dependent recombination (HDR). A consideration of this approach will be whether transgenic expression of multiple different B-linked sequences simultaneously is required to cause a phenotype of interest. Despite these obstacles, genome editing provides a very promising means for finally understanding how B chromosomes mediate their own transmission and drive at the mechanistic level.

## Figures and Tables

**Table 1:** Protein-coding genes located on B chromosomes in different species. (NK = not known).

Functional Group	Gene Name	Organism	Transcribed	Gene Integrity	References
**Cell Division and Microtubules**	Tubulin beta-1 (*TUBB1*)	Cichlid fishes	yes	High	[27]
	Tubulin beta-5 (*TUBB5*)	Cichlid fishes	yes	High	[27]
	Spindle and kinetochore-associated protein-1 (*SKA-1*)	Cichlid fishes	yes	High	[27]
	Kinesin-like protein-11 (*KIFF11*)	Cichlid fishes	yes	High	[27]
	Centromere-associated protein-E (*CENP-E*)	Cichlid fishes	yes	High	[27]
	Centromere-associated protein-N (*CENP-N*)	Red fox	NK	NK	[34]
	Cytoskeleton-associated protein 2 (*CKAP2*)	Grasshoppers	yes	truncated	[24]
	Condensin I complex subunit G (*CAP-G*)	Grasshoppers	yes	truncated	[24]
	E3 ubiquitin-protein ligase *MYCBP2*	Grasshoppers	yes	truncated	[24]
	Kinesin-like protein *KIF20A*	Grasshoppers	yes	High	[24]
	DNA topoisomerase 2-alpha (*TOP2A*)	Grasshoppers	yes	truncated	[24]
	Kinesin-3-like	Rye	yes	High	[19,23]
	Shortage in chiasmata gene (*SHOC 1*)	Rye	yes	High	[19,23]
	Chromosome-associated kinesin *KIF4A*-like	Rye	yes	high + truncated	[19,23]
	Aurora kinase-B (*AURK*)	Cichlid fishes	yes	High	[27]
	Separin-like protein	Cichlid fishes	yes	High	[27]
	Coiled-coil and C2 domain Containing 2A (*CC2D2A*)	Deer	NK	NK	[34]
	Ecotropic viral integration site 5-like (*EVI5*)	Deer	NK	NK	[34]
	E3 ubiquitin-protein ligase *CHFR*	Deer	NK	NK	[34]
	G1/S-specific cyclin-D2 (*CCND2*)	Deer	NK	NK	[34]
	Tripartite motif-containing 67 (*TRIM67*)	Deer	NK	NK	[34]
	Palladin (*PALLD*)	Deer	NK	NK	[34]
	Cdc42 effector protein 4 (*CDC42EP4*)	Deer	NK	NK	[34]
	v-kit Hardy-Zuckerman 4 feline sarcoma viral oncogene homolog (*C-KIT*)	Red fox	NK	NK	[34]
		Raccoon dogs	NK	NK	[34]
		Deer	NK	NK	[34]
**Differentiation, Proliferation**	Kinase insert domain receptor (*KDR*)	Raccoon dogs	NK	NK	[34]
	Low density lipoprotein receptor-related protein 1B(*LRP1B*)	Raccoon dogs	NK	NK	[29,34]
	*AICDA*	Raccoon dogs	NK	NK	[34]
	*RET*	Raccoon dogs	NK	NK	[34]
	*APOBEC1*	Raccoon dogs	NK	NK	[34]
	*ARNTL*	Raccoon dogs	NK	NK	[34]
	*BARX2*	Raccoon dogs	NK	NK	[34]
	*BTBD10*	Raccoon dogs	NK	NK	[34]
	*COL4A3BP*	Raccoon dogs	NK	NK	[34]
	*CXCR4*	Raccoon dogs	NK	NK	[34]
	*ENPP1*	Raccoon dogs	NK	NK	[34]
	*GDF3*	Raccoon dogs	NK	NK	[34]
	*GNAS*	Raccoon dogs	NK	NK	[34]
	*HMGCR*	Raccoon dogs	NK	NK	[34]
	*JAG1*	Raccoon dogs	NK	NK	[34]
	*MDM4*	Raccoon dogs	NK	NK	[34]
	*TNNI3K*	Deer	NK	NK	[34]
	*ZNF268*	Deer	NK	NK	[34]
	*ACVR2B*	Deer	NK	NK	[34]
	*BCL6*	Deer	NK	NK	[34]
	*BST1*	Deer	NK	NK	[34]
	*CD38*	Deer	NK	NK	[34]
	*DHCR7*	Deer	NK	NK	[34]
	*DLEC1*	Deer	NK	NK	[34]
	*EOMES*	Deer	NK	NK	[34]
	*FBXL5*	Deer	NK	NK	[34]
	*FGFBP1*	Deer	NK	NK	[34]
	*FNIP1*	Deer	NK	NK	[34]
	*GABRB1*	Deer	NK	NK	[34]
	*GFI1*	Deer	NK	NK	[34]
	*HPSE*	Deer	NK	NK	[34]
	*MYD88*	Deer	NK	NK	[34]
	*PLCD1*	Deer	NK	NK	[34]
	*SDK2*	Deer	NK	NK	[34]
	*SERPINB9*	Deer	NK	NK	[34]
	*SSBP3*	Deer	NK	NK	[34]
	*SST*	Deer	NK	NK	[34]
	*SSTR2*	Deer	NK	NK	[34]
	*TXK*	Deer	NK	NK	[34]
	*CIP2A* (*CIP2A* protein)	Grasshopper	yes	High	[34]
**Neuron Synapse, Cell Junction**	Cadherin-associated protein-2 (*CTNND2*)	Red Fox	NK	NK	[34]
	*LRRC7*	Raccoon dogs	NK	NK	[34]
	*CXCR4*	Raccoon dogs	NK	NK	[34]
	*ARHGAP32*	Raccoon dogs	NK	NK	[34]
	*SDK1* and *2*	Deer	NK	NK	[34]
	*GABRA4* and *GABRB1*	Deer	NK	NK	[34]
	*LPP*	Deer	NK	NK	[34]
	*SHANK2*	Deer	NK	NK	[34]
**Recombination and Repair**	DNA repair protein *XRCC2*	Cichlid fishes	yes	High	[27]
	SC protein-2 (*SYCP-2*)	Cichlid fishes	yes	High	[27]
	Regulator of telomere elongation helicase (*RTEL*)	Cichlid fishes	ye	High	[27]
**Regulation of Transcrption**	Peroxisome proliferator-activated receptor gamma coactivator-1 (*PPRC1*)	Cichlid fishes	yes	Low	[27]
	Mesogenin-1 (*MSGN1*)	Cichlid fishes	yes	Low	[28]
	C-Myc-binding protein (*MYCBP*)	Cichlid fishes	yes	Low	[28]
	Nuclear receptor-subfamily 2-group F-member 6 (*NR2F6*)	Cichlid fishes	yes	Low	[28]
	Zinc finger protein-596 (*ZNF596*)	Cichlid fishes	yes	High	[28]
	DEAD-box ATP-dependent RNA helicase 7	Maize	yes	High	[48]
	Myb-like DNA-binding domain	Maize	yes	High	[48]
	Conserved mid region of cactin	Maize	yes	High	[48]
	Argonaute-likeprotein (*AGO4*)	Rye	yes	High	[23]
	DNA (cytosine-5-)-methyltransferase	Rye	yes	High	[19]
	Ubiquitin ligase sinat5	Rye	yes	High	[19]
	histone-lysine n-methyltransferase	Rye	yes	High	[19]
	protein kinase subfamily lrk10l-2	Rye	yes	High	[19]
**Sex determination and Differentiation**	Wilms tumor gene	cichlid fishes	yes	Low	[27]
	pre-B-cell leukemia transcription factor 1	cichlid fishes	yes	Low	[27]
	*FKBP4*	cichlid fishes	yes	Low	[27]
	*FNDC3A*	cichlid fishes	yes	Low	[27]
**Metabolism Regulation**	Fucose-1-phosphate guanylyltransferase (*FPGT*)	Siberian Roe deer	yes	High	[49]
		Raccoon dogs	NK	NK	[29,34]
	Lysosomal alpha-mannosidase	Raccoon dogs	NK	NK	[29,34]
	Hydroxypyruvate isomerase (*HYI*)	Grasshoppers	yes	truncated	[24]
	Putative aldose reductase-related protein	Maize	yes	High	[48]
	Leucine-rich repeat- containing protein 23 (*LRC23*)	Cichlid fishes	yes	Low	[27,28]
**Leucin-Rich Protein**	Acidic leucine-rich nuclear phosphoprotein 32 family member E (Cpd1)	Cichlid fishes	yes	High	[27]
	Leucine-rich repeats and immunoglobulin-like domains 1(*LRIG1*)	Raccoon dogs	NK	NK	[29,34]
	Leucine-rich repeat and IQ domain-containing protein 3 (*LRRIQ3*)	Siberian Roe deer	yes	High	[49]
**Olfactory Receptors**	Olfactory receptors 5F1 (or *OR11-10*)	Cichlid fishes	yes	High	[27]
	Olfactory receptor 6C4 (or *OR12-10*	Cichlid fishes	yes	High	[27]
	Olfactory receptor 6N1 (or *OR6N1*)	Cichlid fishes	yes	High	[27]
	Olfactory receptor 51E1 (or *OR51E1*	Cichlid fishes	yes	High	[27]
**Ribonucleotide Binding**	GTP-binding protein 6 (*GTPB6*)	Grasshoppers	yes	High	[24]
	Mitochondrial GTPase 1 (*MTG1*)	Grasshoppers	yes	High	[24]
**Development**	Indian hedgehog homolog b (*IHHB*)	Raccoon dogs	NK	NK	[29]
**Immune Responses**	Rnasel 2 (Ribonuclease-like 2)	Raccoon dogs	NK	NK	[29]
**Cell-cell Signalling and Cellular Response to Stimuli**	*VPS10* domain receptor protein *SORCS 3*–like	Raccoon dogs	NK	NK	[29]
	*SLIT*	Grasshoppers	yes	truncated	[34]
**Histones**	*H3* and *H4*	Migratory locust	NK	it varies among copies	[52]
		Grasshoppers	NK	NK	[53]

## References

[1] Jones R.N. (1991). B-Chromosome drive. Am. Nat..

[2] Jones R.N. (1995). B chromosomes in plants. New Phytol..

[3] Camacho J.P., Sharbel T.F., Beukeboom L.W. (2000). B-chromosome evolution. Philos. Trans. R. Soc. Lond. B. Biol. Sci..

[4] Werren J.H., Stouthamer R. (2003). PSR (paternal sex ratio) chromosomes: the ultimate selfish genetic elements. Genetica.

[5] Hurst G.D.D., Werren J.H. (2001). The role of selfish genetic elements in eukaryotic evolution. Nat. Rev. Genet..

[6] Hewitt G.M., John B. (1967). The B-chromosome system of *Myrmeleotettix macculatus* (Thunb.). Chromosoma.

[7] Fontana P.G., Vickery V.R. (1973). Segregation-distortion in the B-chromosome system of *Tettigidea lateralis* (Say) (Orthoptera: Tetrigidae). Chromosoma.

[8] Kimura M., Kayano H. (1961). The maintenance of super-numerary chromosomes in wild populations of *Lilium callosum* by preferential segregation. Genetics.

[9] Banaei-Moghaddam A.M., Schubert V., Kumke K., Weiβ O., Klemme S., Nagaki K., Macas J., González-Sánchez M., Heredia V., Gómez-Revilla D. (2012). Nondisjunction in favor of a chromosome: The mechanism of rye B chromosome drive during pollen mitosis. Plant Cell.

[10] Han F., Lamb J.C., Yu W., Gao Z., Birchler J.A. (2007). Centromere function and nondisjunction are independent components of the maize B chromosome accumulation mechanism. Plant Cell.

[11] Müntzing A. (2010). Chromosome number, nuclear volume and pollen grain size in *Galeopsis*. Hereditas.

[12] Reed K.M. (1993). Cytogenetic analysis of the paternal sex ratio chromosome of *Nasonia vitripennis*. Genome.

[13] Reed K.M., Werren J.H. (1995). Induction of paternal genome loss by the paternal-sex-ratio chromosome and cytoplasmic incompatibility bacteria (Wolbachia): A comparative study of early embryonic events. Mol. Reprod. Dev..

[14] Beukeboom L.W., Werren J.H. (1993). Deletion analysis of the selfish B chromosome, paternal sex ratio (PSR), in the parasitic wasp *Nasonia vitripennis*. Genetics.

[15] Klemme S., Banaei-Moghaddam A.M., Macas J., Wicker T., Novák P., Houben A. (2013). High-copy sequences reveal distinct evolution of the rye B chromosome. New Phytol..

[16] Navarro-Domínguez B., Martín-Peciña M., Ruiz-Ruano F.J., Cabrero J., Corral J.M., López-León M.D., Sharbel T.F., Camacho J.P.M. (2019). Gene expression changes elicited by a parasitic B chromosome in the grasshopper *Eyprepocnemis plorans* are consistent with its phenotypic effects. Chromosoma.

[17] Navarro-Domínguez B., Ruiz-Ruano F.J., Camacho J.P.M., Cabrero J., López-León M.D. (2017). Transcription of a B chromosome CAP-G pseudogene does not influence normal Condensin Complex genes in a grasshopper. Sci. Rep..

[18] Carchilan M., Kumke K., Mikolajewski S., Houben A. (2009). Rye B chromosomes are weakly transcribed and might alter the transcriptional activity of A chromosome sequences. Chromosoma.

[19] Banaei-Moghaddam A.M., Meier K., Karimi-Ashtiyani R., Houben A. (2013). Formation and expression of pseudogenes on the B chromosome of rye. Plant Cell.

[20] Delgado M., Caperta A., Ribeiro T., Viegas W., Jones R.N., Morais-Cecílio L. (2004). Different numbers of rye B chromosomes induce identical compaction changes in distinct A chromosome domains. Cytogenet. Genome Res..

[21] Rubtsov N.B., Borisov Y.M. (2018). Sequence composition and evolution of mammalian B chromosomes. Genes.

[22] Ward E.J. (1973). Nondisjunction: localization of the controlling site in the maize B chromosome. Genetics.

[23] Ma W., Gabriel T.S., Martis M.M., Gursinsky T., Schubert V., Vrána J., Doležel J., Grundlach H., Altschmied L., Scholz U. (2016). Rye B chromosomes encode a functional Argonaute-like protein within vitroslicer activities similar to its A chromosome paralog. New Phytol..

[24] Navarro-Domínguez B., Ruiz-Ruano F.J., Cabrero J., Corral J.M., López-León M.D., Sharbel T.F., Camacho J.P.M. (2017). Protein-coding genes in B chromosomes of the grasshopper *Eyprepocnemis plorans*. Sci. Rep..

[25] Akbari O.S., Antoshechkin I., Hay B.A., Ferree P.M. (2013). Transcriptome profiling of *Nasonia vitripennis* testis reveals novel transcripts expressed from the selfish B chromosome, paternal sex ratio. G3.

[26] Li Y., Jing X.A., Aldrich J.C., Clifford C., Chen J., Akbari O.S., Ferree P.M. (2017). Unique sequence organization and small RNA expression of a “selfish” B chromosome. Chromosoma.

[27] Valente G.T., Conte M.A., Fantinatti B.E.A., Cabral-de-Mello D.C., Carvalho R.F., Vicari M.R., Kocher T.D., Martins C. (2014). Origin and evolution of B chromosomes in the cichlid fish *Astatotilapia latifasciata* based on integrated genomic analyses. Mol. Biol. Evol..

[28] Valente G.T., Nakajima R.T., Fantinatti B.E.A., Marques D.F., Almeida R.O., Simões R.P., Martins C. (2017). B chromosomes: from cytogenetics to systems biology. Chromosoma.

[29] Becker S.E.D., Thomas R., Trifonov V.A., Wayne R.K., Graphodatsky A.S., Breen M. (2011). Anchoring the dog to its relatives reveals new evolutionary breakpoints across 11 species of the Canidae and provides new clues for the role of B chromosomes. Chromosome Res..

[30] Coleman J.J., Rounsley S.D., Rodriguez-Carres M., Kuo A., Wasmann C.C., Grimwood J., Schmutz J., Taga M., White G.J., Zhou S. (2009). The genome of *Nectria haematococca*: contribution of supernumerary chromosomes to gene expansion. PLoS Genet..

[31] Goodwin S.B., M’barek S.B., Dhillon B., Wittenberg A.H.J., Crane C.F., Hane J.K., Foster A.J., Van der Lee T.A.J., Grimwood J., Aerts A. (2011). Finished genome of the fungal wheat pathogen *Mycosphaerella graminicola* reveals dispensome structure, chromosome plasticity, and stealth pathogenesis. PLoS Genet..

[32] Houben A., Banaei-Moghaddam A.M., Klemme S., Timmis J.N. (2014). Evolution and biology of supernumerary B chromosomes. Cell. Mol. Life Sci..

[33] Martis M.M., Klemme S., Banaei-Moghaddam A.M., Blattner F.R., Macas J., Schmutzer T., Scholz U., Gundlach H., Wicker T., Simkova H. (2012). Selfish supernumerary chromosome reveals its origin as a mosaic of host genome and organellar sequences. Proc. Natl. Acad. Sci..

[34] Makunin A., Romanenko S., Beklemisheva V., Perelman P., Druzhkova A., Petrova K., Prokopov D., Chernyaeva E., Johnson J., Kukekova A. (2018). Sequencing of supernumerary chromosomes of red fox and raccoon dog confirms a non-random gene acquisition by B Chromosomes. Genes.

[35] Ruban A., Schmutzer T., Scholz U., Houben A. (2017). How next-generation sequencing has aided our understanding of the sequence composition and origin of B chromosomes. Genes.

[36] Cheng Y.-M., Lin B.-Y. (2003). Cloning and characterization of maize B chromosome sequences derived from microdissection. Genetics.

[37] Bugrov A.G., Karamysheva T.V., Perepelov E.A., Elisaphenko E.A., Rubtsov D.N., Warchałowska-Śliwa E., Tatsuta H., Rubtsov N.B. (2007). DNA content of the B chromosomes in grasshopper Podisma kanoi Storozh. (Orthoptera, Acrididae). Chromosome Res..

[38] Ruiz-Ruano F.J., Cabrero J., López-León M.D., Sánchez A., Camacho J.P.M. (2018). Quantitative sequence characterization for repetitive DNA content in the supernumerary chromosome of the migratory locust. Chromosoma.

[39] Coan R., Martins C. (2018). Landscape of transposable elements focusing on the B chromosome of the cichlid fish *Astatotilapia latifasciata*. Genes.

[40] Marques A., Klemme S., Houben A. (2018). Evolution of plant B chromosome enriched sequences. Genes.

[41] McAllister B.F. (1995). Isolation and characterization of a retroelement from B chromosome (PSR) in the parasitic wasp *Nasonia vitripennis*. Insect Mol. Biol..

[42] McAllister B.F., Werren J.H. (1997). Hybrid origin of a B chromosome (PSR) in the parasitic wasp *Nasonia vitripennis*. Chromosoma.

[43] Perfectti F., Werren J.H. (2001). The interspecific origin of B chromosomes: experimental evidence. Evolution.

[44] McVean G.T. (1995). Fractious chromosomes: hybrid disruption and the origin of selfish genetic elements. Bioessays.

[45] Schartl M., Nanda I., Schlupp I., Wilde B., Epplen J.T., Schmid M., Parzefall J. (1995). Incorporation of subgenomic amounts of DNA as compensation for mutational load in a gynogenetic fish. Nature.

[46] Banaei-Moghaddam A.M., Martis M.M., Macas J., Gundlach H., Himmelbach A., Altschmied L., Mayer K.F.X., Houben A. (2015). Genes on B chromosomes: Old questions revisited with new tools. Biochim. Biophys. Acta.

[47] Palazzo A.F., Lee E.S. (2015). Non-coding RNA: what is functional and what is junk?. Front. Genet..

[48] Huang W., Du Y., Zhao X., Jin W. (2016). B chromosome contains active genes and impacts the transcription of A chromosomes in maize (*Zea mays* L.). BMC Plant Biol..

[49] Trifonov V.A., Dementyeva P.V., Larkin D.M., O’Brien P.C.M., Perelman P.L., Yang F., Ferguson-Smith M.A., Graphodatsky A.S. (2013). Transcription of a protein-coding gene on B chromosomes of the Siberian roe deer (*Capreolus pygargus*). BMC Biol..

[50] Gaudet P., Livstone M.S., Lewis S.E., Thomas P.D. (2011). Phylogenetic-based propagation of functional annotations within the Gene Ontology consortium. Brief. Bioinform..

[51] Malik H.S., Bayes J.J. (2006). Genetic conflicts during meiosis and the evolutionary origins of centromere complexity. Biochem. Soc. Trans..

[52] Teruel M., Cabrero J., Perfectti F., Camacho J.P.M. (2010). B chromosome ancestry revealed by histone genes in the migratory locust. Chromosoma.

[53] Oliveira N.L., Cabral-de-Mello D.C., Rocha M.F., Loreto V., Martins C., Moura R.C. (2011). Chromosomal mapping of rDNAs and H3 histone sequences in the grasshopper *Rhammatocerus brasiliensis* (*Acrididae, gomphocerinae*): extensive chromosomal dispersion and co-localization of 5S rDNA/H3 histone clusters in the A complement and B chromosome. Mol. Cytogenet..

[54] SanMiguel P., Tikhonov A., Jin Y.K., Motchoulskaia N., Zakharov D., Melake-Berhan A., Springer P.S., Edwards K.J., Lee M., Avramova Z. (1996). Nested retrotransposons in the intergenic regions of the maize genome. Science.

[55] Tang W., Mun S., Joshi A., Han K., Liang P. (2018). Mobile elements contribute to the uniqueness of human genome with 15,000 human-specific insertions and 14 Mbp sequence increase. DNA Res..

[56] Cheng Y.-M., Lin B.-Y. (2004). Molecular organization of large fragments in the maize B chromosome: indication of a novel repeat. Genetics.

[57] Ziegler C.G., Lamatsch D.K., Steinlein C., Engel W., Schartl M., Schmid M. (2003). The giant B chromosome of the cyprinid fish *Alburnus alburnus harbours* a retrotransposon-derived repetitive DNA sequence. Chromosome Res..

[58] Gross L. (2006). Transposon silencing keeps jumping genes in their place. PLoS Biol..

[59] McGurk M.P., Barbash D.A. (2018). Double insertion of transposable elements provides a substrate for the evolution of satellite DNA. Genome Res..

[60] Ramos É., Cardoso A.L., Brown J., Marques D.F., Fantinatti B.E.A., Cabral-de-Mello D.C., Oliveira R.A., O’Neill R.J., Martins C. (2016). The repetitive DNA element BncDNA, enriched in the B chromosome of the cichlid fish *Astatotilapia latifasciata*, transcribes a potentially noncoding RNA. Chromosoma.

[61] Perry R.B.-T., Ulitsky I. (2016). The functions of long noncoding RNAs in development and stem cells. Development.

[62] Ulitsky I., Bartel D.P. (2013). lincRNAs: genomics, evolution, and mechanisms. Cell.

[63] Park Y., Oh H., Meller V.H., Kuroda M.I. (2005). Variable splicing of non-coding roX2 RNAs influences targeting of MSL dosage compensation complexes in *Drosophila*. RNA Biol..

[64] Park Y. (2002). Extent of chromatin spreading determined by roX RNA Recruitment of MSL proteins. Science.

[65] Lucchesi J.C., Kuroda M.I. (2015). dosage compensation in *Drosophila*. Cold Spring Harb. Perspect. Biol..

[66] Ilik I.A., Quinn J.J., Georgiev P., Tavares-Cadete F., Maticzka D., Toscano S., Wan Y., Spitale R.C., Luscombe N., Backofen R. (2013). Tandem stem-loops in roX RNAs act together to mediate X chromosome dosage compensation in *Drosophila*. Mol. Cell.

[67] Sahakyan A., Yang Y., Plath K. (2018). The Role of Xist in X-chromosome dosage compensation. Trends Cell Biol..

[68] Rougeulle C., Chaumeil J., Sarma K., Allis C.D., Reinberg D., Avner P., Heard E. (2004). Differential histone H3 Lys-9 and Lys-27 methylation profiles on the X chromosome. Mol. Cell. Biol..

[69] Han P., Chang C.-P. (2015). Long non-coding RNA and chromatin remodeling. RNA Biol..

[70] Böhmdorfer G., Wierzbicki A.T. (2015). Control of chromatin structure by long noncoding RNA. Trends Cell Biol..

[71] Aldrich J.C., Leibholz A., Cheema M.S., Ausiό J., Ferree P.M. (2017). A “selfish” B chromosome induces genome elimination by disrupting the histone code in the jewel wasp *Nasonia vitripennis*. Sci. Rep..

[72] Zhang C. (2009). Novel functions for small RNA molecules. Curr. Opin. Mol. Ther..

[73] Chapman E.J., Carrington J.C. (2007). Specialization and evolution of endogenous small RNA pathways. Nat. Rev. Genet..

[74] López-León M.D., Cabrero J., Pardo M.C., Viseras E., Camacho J.P.M., Santos J.L. (1993). Generating high variability of B chromosomes in *Eyprepocnemis plorans* (grasshopper). Heredity.

